# Association between Inflammatory Bowel Disease and Iridocyclitis: A Mendelian Randomization Study

**DOI:** 10.3390/jcm12041282

**Published:** 2023-02-06

**Authors:** Yang Meng, Zongbiao Tan, Chuan Liu, Weiguo Dong, Changzheng Chen

**Affiliations:** 1 Department of Ophthalmology, Renmin Hospital of Wuhan University, 238 Jiefang Road, Wuhan 430060, China; 2 Department of Gastroenterology, Renmin Hospital of Wuhan University, 238 Jiefang Road, Wuhan 430060, China

**Keywords:** inflammatory bowel disease, ulcerative colitis, Crohn’s disease, iridocyclitis, Mendelian randomization

## Abstract

Background: Iridocyclitis (IC) is a common extraintestinal manifestation of inflammatory bowel disease (IBD). Observational studies showed patients with ulcerative colitis (UC) and Crohn’s disease (CD) both have a higher risk of IC. However, due to the inherent limitations of observational studies, the association and its directionality between the two forms of IBD and IC remain undiscerned. Methods: Genetic variants for IBD and IC were selected as instruments from genome-wide association studies (GWAS) and FinnGen database as instrumental variables, respectively. A bidirectional Mendelian randomization (MR) and multivariable MR were performed successively. Three different MR methods were performed to determine the causal association, including inverse-variance weighted (IVW), MR Egger, and weighted median, whereas IVW was used as the main analysis. Different methods for sensitivity analysis were used, including MR-Egger intercept test, MR Pleiotropy RESidual Sum and Outlier test, Cochran’s Q test, and leave-one-out analysis. Results: Bidirectional MR suggested both UC and CD were positively associated with IC as a whole, acute and subacute IC, and chronic IC. However, in the MVMR analysis, only the association from CD to IC remained stable. In the reverse analysis, no association was observed from IC to UC or CD. Conclusions: Both UC and CD are associated with an increased risk of IC compared with healthy individuals. However, the association between CD and IC is stronger. In the reverse direction, patients with IC do not suffer a higher risk of UC or CD. We emphasize the importance of ophthalmic examinations for IBD patients, especially for CD patients.

## 1. Introduction

Inflammatory bowel disease (IBD), including ulcerative colitis (UC) and Crohn’s disease (CD), is a chronic and systemic disease mainly characterized by relapsing inflammation in the gastrointestinal tract with accompanying extraintestinal manifestations and immune dysfunction [1,2,3]. Although substantial progress has been made in the treatment of IBD (biologic agents), the pathogenesis of IBD has not been fully understood. As a disease of affluence, the prevalence of IBD is increasing globally, delivering a high disease burden worldwide [4].

Iridocyclitis (IC) is the most common type of uveitis, occurring in up to 17% of IBD patients [5,6]. Patients with IC may experience blurred vision, eye pain, redness, and photophobia [5]. Usually, IC is a benign disease, but if left unrecognized and untreated, serious consequences such as cataracts and secondary glaucoma may also happen, both of which may lead to blindness [5,7]. As an extraintestinal manifestation of IBD, IC may parallel the intestinal disease activity, and is more frequently seen with prolonged disease duration, considerably affecting the quality of life of IBD patients [8]. Two large cohort studies suggested IC is one of the most common immune-mediated inflammatory diseases in IBD population, with an odds–ratio of 3.2 [95%CI: 2.0–5.4] compared with the control groups [9,10]. Exploring the association between the two diseases will help to advance the understanding of IBD and IC and may yield new insights into early diagnosis of IBD and IC. However, since many patients in the cohort studies were diagnosed with IC before IBD, as well as the existence of reverse causation and confounders, the association and its directionality between IBD and IC remain undiscerned.

Mendelian randomization (MR) can overcome these defects by using genetic variants identified in genome-wide association studies (GWAS) as instrumental variables (IVs) for an exposure to infer whether the exposure causally affects an outcome [11]. MR depends on the random allocation of genetic variants (IVs) at conception, namely the law of independent assortment proposed by Gregor Mendel, which causes a random distribution of these genetic variants in a population [11,12]. An individual either carries specific exposure-associated genetic variants or not, both of which are naturally determined before birth [13]. Usually, these variants are not subjected to confounders or reverse causation, thus the differences in outcome between individuals who carry these variants and those who do not are resulted from the difference in the risk factor [12,13]. This, together with the convenient availability of the large number of potential IVs to represent interested exposures, make MR a time- and cost-saving method to detect potential associations between exposures and outcomes.

In this present study, we conducted a bidirectional MR and multivariable MR (MVMR) successively using data from GWAS on IBD and IC to investigate the potential bidirectional association between IBD (including both UC and CD) and IC.

## 2. Materials and Methods

**Figure 1:**
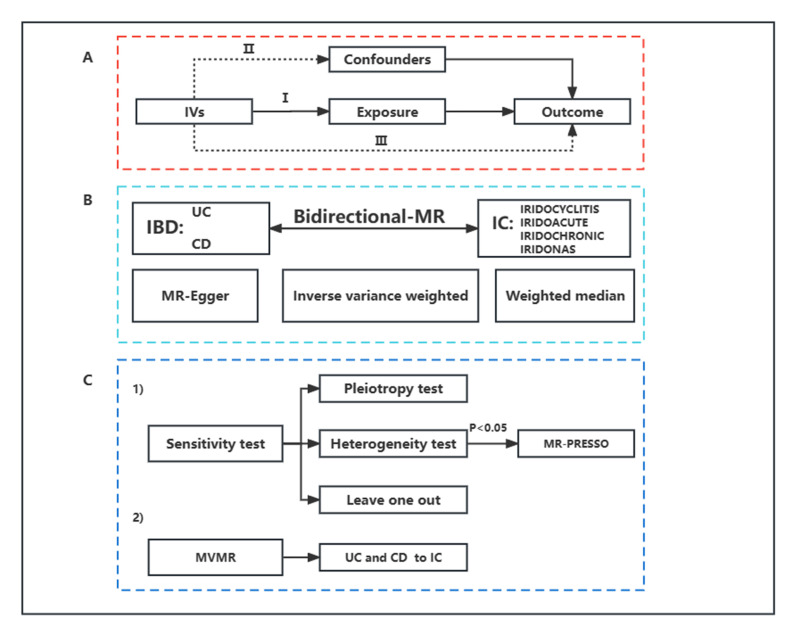
An overview of the study design. (**A**) Illustration of MR. MR is based on three principal assumptions: (Ⅰ) the IVs are associated with the exposure; (Ⅱ). the IVs are not associated with confounders; (Ⅲ). the IVs influence the outcome only through no other ways but exposure. (**B**) Illustration of bidirectional MR. When IBD was set as exposure, IC was the outcome. When IC was set as exposure, IBD was the outcome. Three MR methods were performed to assess the causal estimations between IBD and IC, including inverse-variance weighted, MR Egger, and weighted median, whereas the first was used as the main analysis. IBD data included UC and CD. IC data included IRIDOCYCLITIS (IC as a whole), IRIDOACUTE (acute and subacute IC), IRIDOCHRONIC (chronic IC), IRIDONAS (other and unspecified IC). (**C**) Illustration of sensitivity analysis and MVMR. (1): MR-Egger intercept test was used to assess pleiotropy. Cochran’s Q test was used to detect heterogeneity. MR-PRESSO test was used to detect and remove outliers when heterogeneity is found. Leave-one-out analysis was used to check the potential influence of the IVs on MR estimates. (2): To avoid the confounders between UC and CD, a further MVMR analysis was performed. MR: Mendelian randomization; IVs: instrumental variables; IBD: Inflammatory bowel disease; UC: ulcerative colitis; CD: Crohn’s disease; IC: iridocyclitis; MR-PRESSO: MR Pleiotropy RESidual Sum and Outlier; MVMR: multivariable MR.

This is a bidirectional two-sample MR and MVMR study. An overview of the study design is presented in Figure 1. Since this study re-analyzed previously collected and published data, no additional ethical approval was required.

### 2.1. Data Sources

#### 2.1.1. Data Sources for IBD

The IBD data, including UC and CD, were obtained from the IEU Open GWAS database. The IBD data included 25,042 cases and 34,915 controls, the UC data included 12,366 cases and 33,609 controls, and the CD data included 12,194 cases and 28,072 controls [14]. These diagnoses were based on endoscopic, radiological, and histopathological criteria that were widely accepted.

#### 2.1.2. Data Sources for IC

IC data were extracted from the FinnGen database (http://www.finngen.fi) (accessed on 5 December 2022) [15], which was the version published in December 2022. Population selection was based on the International Classification of Diseases-10 (ICD-10). IC population included IC as a whole (IRIDOCYCLITIS; ICD-10 H20; 6349 cases and 325,475 controls), acute and subacute IC (IRIDOACUTE; ICD-10 H20.0; 5405 cases and 325,475 controls), chronic IC (IRIDOCHRONIC; ICD-10 H20.1; 1202 cases and 32,5475 controls), other and unspecified IC (IRIDONAS; ICD-10 H20.8 and H20.9; 891 cases and 325,475 controls).

### 2.2. Selection of Instrumental Variables (Genetic Instruments)

To identify the causal association between IBD (including UC and CD) and IC, we first screened instrumental variables indicating IBD and IC. Instrumental variables were selected according to the following criteria: (1) single nucleotide polymorphism (SNP) with GWAS-correlated *p*-value < 5 × 10^−8^ so that it was strongly related to the exposure; (2) linkage disequilibrium (LD) r^2^ < 0.001, and <1000 KB from the index variant, with one exception: the threshold of LD r^2^ was relaxed to 0.01 for IRIDOCHRONIC to ensure the number of SNPs analyzed. Additionally, outcome-related SNPs with a threshold of *p*-value < 5 × 10^−8^ would be excluded. Moreover, F-value was calculated to quantify the strength of the instrumental variables. The formula is as follows: F = (beta/SE)^2^, where beta referred to the effect value of the exposure and SE referred to the standard error for effect values of exposure. SNPs with an F-value > 10 were considered sufficient while those with an F-value < 10 were excluded. Finally, we checked in Phenoscanner (www.phenoscanner.medschl.cam.ac.uk) (accessed on 5 December 2022), a large database of genotype and phenotype associations in humans, to detect if the SNPs were associated with the potential confounders [16]. However, since UC and CD did have some shared gene loci, we could not exclude all SNPs that were closely related to both UC and CD. Thus, we further conducted an MVMR to remove the mutual bias between UC and CD.

MVMR is a recently developed method that retains the advantages of conventional MR, such as using instrumental variables to make causal inferences to avoid potential bias, while allowing for simultaneous analysis of the causality of multiple separate but correlated exposures on one outcome [17,18]. In this paper, we conducted an additional MVMR analysis where UC and CD were two separate, but correlated, exposures and IC was the outcome, thus avoiding the confounders between the UC and CD as much as possible.

### 2.3. MR Analyses

Three different MR methods were performed to determine the causal association between IBD and IC, including inverse–variance weighted (IVW), MR Egger, and weighted median, which were based on different assumptions.

The IVW method, which assumes that there is no directional pleiotropic effect of each SNP, uses a meta-analysis approach to combine the effect size of each SNP calculated by Wald ratios and finally yields an overall causal effect of the exposure on the outcome [19]. The MR–Egger method allows all SNPs to have a pleiotropic effect but requires the SNPs to satisfy the InSIDE (Instrument Strength Independent of Direct Effect) assumption [20]. The slope in MR–Egger regression provides a causal estimate of the exposure on the outcome when the InSIDE assumption is met. Moreover, net pleiotropy is indicated by the intercept of MR–Egger regression. The weighted median method can be used to provide a causal estimate in the event that more than half of the instrumental variables are valid in MR analysis [21]. IVW was used as the main analysis whereas MR–Egger and weighted median were used to help improve the interpretation of MR results, despite the latter two methods being usually less efficient.

Moreover, to further justify the MR results, we used different methods for sensitivity analysis. The MR–Egger intercept test was used to assess horizontal pleiotropy. The MR Pleiotropy RESidual Sum and Outlier (MR-PRESSO) test was used to detect outliers. MR-PRESSO has three components: (1) horizontal pleiotropy detection; (2) horizontal pleiotropy correction through removing outlier SNPs, and (3) testing of significant differences in the causal estimates before and after outlier SNPs removal [22]. Then the MR–Egger intercept test was conducted again for horizontal pleiotropy detection. We further performed Cochran’s Q test to detect heterogeneity among all SNPs. Finally, leave-one-out (LOO) analysis was used for sensitivity analysis by excluding each SNP at a time sequentially to check the potential influence of the SNP on an MR estimate.

### 2.4. Statistics

All MR analyses were performed using the “TwoSampleMR” and “MRPRESSO” packages in R statistical software (version 4.1.3). The results of MR analysis were presented in the form of odds ratios (OR) with 95% confidence intervals (CI) to quantify the association between exposure and outcome. Due to multiple testing between IBD (UC and CD) and IC (IRIDOACUTE, IRIDOACUTE, IRIDOCHRONIC, and IRIDONAS), the MR analysis results to determine the causal effect of IBD on IC were only considered statistically significant when Bonferroni corrected *p*-value < 0.0021 (0.05/24). In the process of MVMR from IBD to IC, the threshold for significance was set at Bonferroni corrected *p*-value < 0.0063 (0.05/8).

## 3. Results

### 3.1. Instrumental Variables for IBD (Including UC and CD) and IC

In total, 117 index SNPs were identified as potential genetic IVs for IBD, 89 SNPs were identified for CD, and 62 SNPs were identified for UC, respectively. Detailed information on these SNPs is listed in Supplementary Table S1. After harmonization and removal of palindromic SNPs with intermediate allele frequencies, the remaining SNPs were included in the MR analysis.

26 SNPs were screened as potential IVs for IRIDOCYCLITIS, 28 SNPs for IRIDOACUTE, 15 SNPs for IRIDOCHRONIC, and 9 SNPs for IRIDONAS, respectively. All of these SNPsare listed in Supplementary Table S2. Similarly, all SNPS need to be reconciled and removed from palindromic SNPS with intermediate allele frequencies before being included in MR Analysis. Then, the MR-PRESSO test was performed to check and remove outlier IVs. MR estimates were re-analyzed after removal of outlier IVs. The F-value for each SNP was higher than 10, indicating little chance of weak instrumental variable bias.

### 3.2. Bidirectional MR from IBD (including UC and CD) to IC

**Figure 2:**
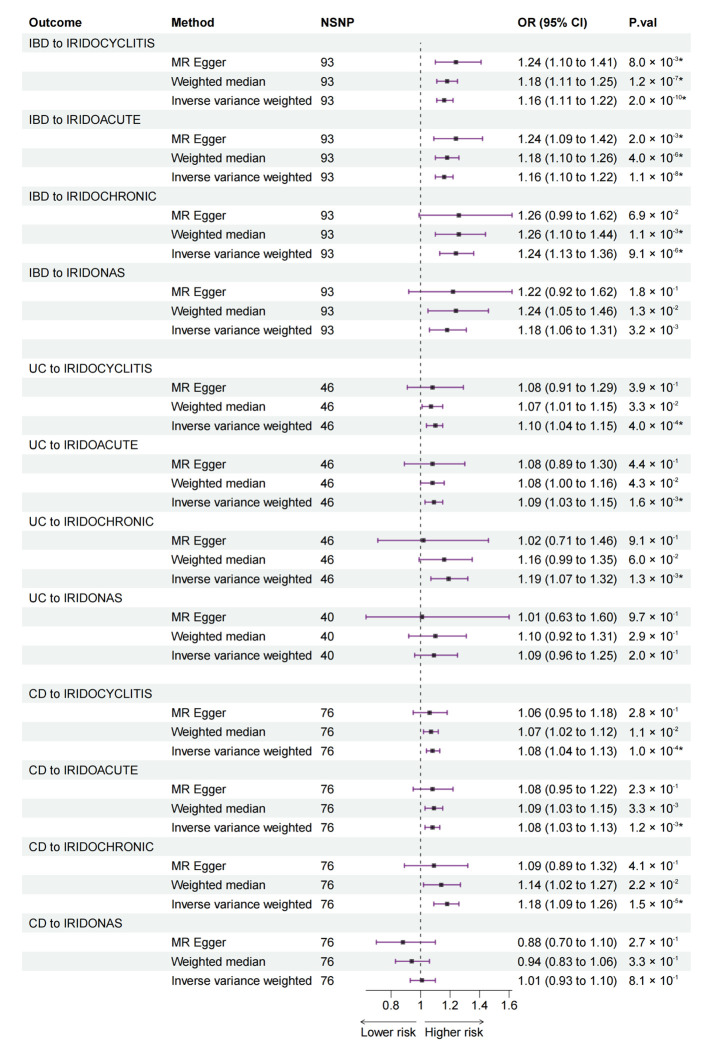
MR estimates from IBD (including UC and CD) to IC in bidirectional MR. MR: Mendelian randomization; IBD: inflammatory bowel disease; UC: ulcerative colitis; CD: Crohn’s disease; IC: iridocyclitis; NSNP: number of single nucleotide polymorphism; OR: odds ratio; CI: confidence interval; IRIDOCYCLITIS: IC as a whole; IRIDOACUTE: acute and subacute IC; IRIDOCHRONIC: chronic IC; IRIDONAS: other and unspecified IC. *: *p*-values of statistical significance (<0.0021) after Bonferroni correction.

The final bidirectional MR results from IBD, UC, and CD to IC are listed in Figure 2.

#### 3.2.1. Bidirectional MR from IBD to IC

The MR estimates from the IVW method showed associations from IBD to IRIDOCYCLITIS (OR: 1.16, 95% CI: 1.11 to 1.22), IRIDOACUTE (OR: 1.16, 95% CI: 1.10 to 1.22), and IRIDOCHRONIC (OR: 1.24, 95% CI: 1.13 to 1.36), whereas no statistical significance from IBD to IRIDONAS was found. Results of the MR–Egger and weighted median methods were either consistent with the IVW method or had no statistical significance (Figure 2).

#### 3.2.2. Bidirectional MR from UC to IC

IVW method revealed associations from UC to IRIDOCYCLITIS (OR: 1.10, 95% CI: 1.04 to 1.15), IRIDOACUTE (OR: 1.09, 95% CI: 1.03 to 1.15), and IRIDOCHRONIC (OR: 1.19, 95% CI: 1.07 to 1.32). MR–Egger and weighted median methods detected no statistical significance for all types of IC (Figure 2).

#### 3.2.3. Bidirectional MR from CD to IC

IVW method suggested associations from CD to IRIDOCYCLITIS (OR: 1.08, 95% CI: 1.04 to 1.13), IRIDOACUTE (OR: 1.08, 95% CI: 1.03 to 1.13), and IRIDOCHRONIC (OR: 1.18, 95% CI: 1.09 to 1.26). MR–Egger and weighted median methods detected no statistical significance (Figure 2).

### 3.3. Bidirectional MR from IC to IBD (Including UC and CD)

**Figure 3:**
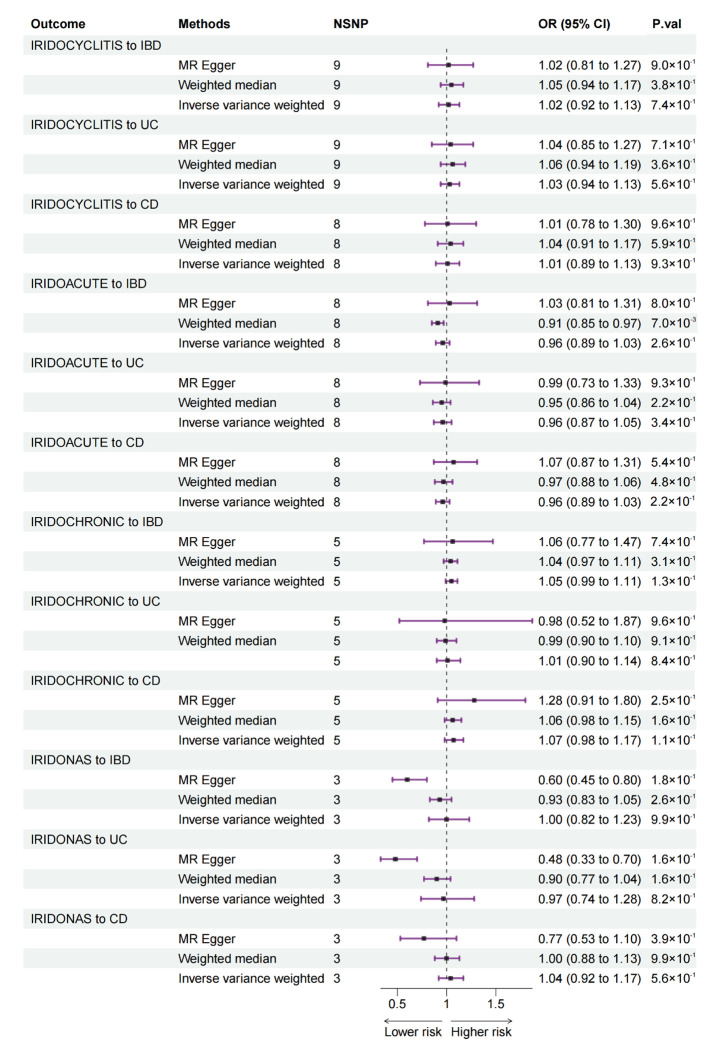
MR estimates from IC to IBD (including UC and CD) in bidirectional MR. MR: Mendelian randomization; IC: iridocyclitis; IBD: inflammatory bowel disease; UC: ulcerative colitis; CD: Crohn’s disease; NSNP: number of single nucleotide polymorphism; OR: odds ratio; CI: confidence interval; IRIDOCYCLITIS: IC as a whole; IRIDOACUTE: acute and subacute IC; IRIDOCHRONIC: chronic IC; IRIDONAS: other and unspecified IC. *p*-values < 0.0021 after Bonferroni correction were considered statistically significant.

Bidirectional MR estimates from the four types of IC to IBD, UC, and CD are listed in Figure 3. In this direction, there was no association from the four types of IC to IBD.

### 3.4. Multivariable MR from UC and CD to IC

**Figure 4:**
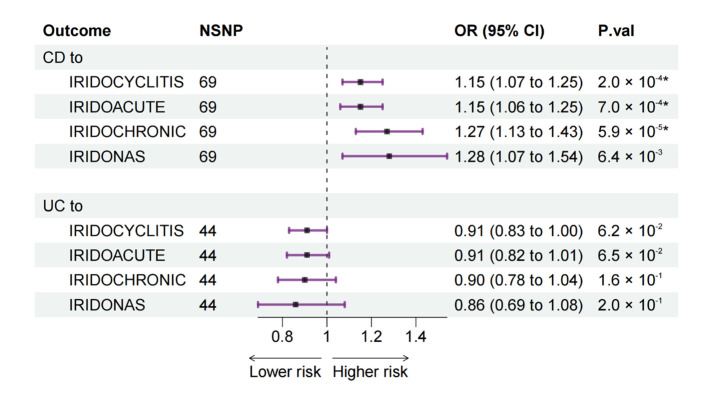
MR estimates from UC and CD to IC in MVMR. MR: Mendelian randomization; UC: ulcerative colitis; CD: Crohn’s disease; IC: iridocyclitis; MVMR: multivariable Mendelian randomization; NSNP: number of single nucleotide polymorphism; OR: odds ratio; CI: confidence interval; IRIDOCYCLITIS: IC as a whole; IRIDOACUTE: acute and subacute IC; IRIDOCHRONIC: chronic IC; IRIDONAS: other and unspecified IC. *: *p*-values of statistical significance (<0.0063) after Bonferroni correction.

According to the results in bidirectional MR, both UC and CD seemed to be associated with three types of IC: IRIDOCYCLITIS, IRIDOACUTE, and IRIDOCHRONIC while not associated with IRIDONAS. Nevertheless, since UC and CD were two interrelated diseases, we further performed an MVMR analysis from UC and CD to IC, to evaluate the direct UC-specific and CD-specific effects on IC (Figure 4). As an extension of conventional MR, MVMR is particularly useful when trying to understand whether two or more related exposures exert a causal effect on the same outcome or when one exposure might be a mediator of other exposures, just like in our study [17,18].

For CD, MVMR results, consistent with bidirectional MR, further confirmed that CD was a risk factor for IRIDOCYCLITIS (OR: 1.15, 95% CI: 1.07 to 1.25), IRIDOACUTE (OR: 1.15, 95% CI: 1.06 to 1.25), and IRIDOCHRONIC (OR: 1.27, 95% CI: 1.13 to 1.43).

For UC, MVMR results showed that UC-specific SNPs seemed not to be related to IC for no statistical significance was observed.

## 4. Discussion

To our knowledge, this is the first study to investigate the potential association and its directionality between IBD and IC. A number of previous observational studies have suggested an increased risk of IC in patients with IBD, including both UC and CD [9,10]. However, observational studies are inevitably affected by potential confounding factors and reverse causality, making causal inferences difficult. Randomized controlled trials (RCTs) provide a solution to these limitations. However, RCTs are much too costly and time-consuming, and in some cases, there may be no appropriate intervention to verify certain hypotheses [12]. As a result, there is no randomized controlled study focused on the relationship between IBD and IC yet. Thus, it remained unclear whether there exists an association between IBD and IC, and, to ask another question, what is the directionality of the association if it does exist? The combination of bidirectional MR and MVMR provides an alternative solution, which is cost- and time-saving.

We first assessed the bidirectional associations between IBD and IC using bidirectional two-sample MR. We found that the genetically predicted risk of IBD as a whole was positively associated with an increased risk of all studied types of IC, whereas UC and CD were positively associated with three types of IC except other and unspecified IC (IRIDONAS) (Figure 2). In the reverse direction, no association was observed from IC to IBD (Figure 3). Then, due to the interrelated nature of UC and CD, a further MVMR analysis was performed to evaluate the direct causal effect of UC and CD on IC separately (Figure 4). MVMR analysis showed that only genetic liability to CD was associated with an increased risk of IC as a whole (IRIDOCYCLITIS), acute and subacute IC (IRIDOACUTE), and chronic IC (IRIDOCHRONIC). However, the MVMR results did not mean that UC is not associated with IC because there were some shared SNPs between UC and CD. These overlapped SNPs played a role in the pathophysiology of both UC and CD.

After synthesizing the results of bidirectional MR and MVMR, we believe that, compared with healthy individuals, patients with IBD (both UC and CD) are more likely to suffer from IC. Notably, although both UC and CD are related to IC, CD has a stronger association with IC. The shared SNPs between UC and CD may explain the relationship between UC and IC as well as CD and IC in bidirectional MR. The MVMR results could only illustrate that CD-specific SNPs were related to IC, whereas UC-specific SNPs seemed not. In the reverse direction, patients with IC do not suffer a higher risk of UC or CD.

So far, a higher prevalence of IC in IBD patients has been reported in several population-based observational investigations [9,10]. Specifically, in a nationwide cohort study in Denmark with 14,377 incident IBD cases, the adjusted incidence rate ratio of IC in patients with CD and UC were 8.24 (95% CI: 3.42 to 19.89) and 3.29 (95% CI: 1.71 to 6.29), respectively [9]. In a larger cross sectional study with 47,325 IBD patients, the odds ratios for IC in CD patients and UC patients were 3.6 (95% CI: 2.7 to 4.7) and 2.4 (95% CI: 2.0 to 2.9), respectively [10]. These findings were consistent with our MR results, that is, although both the subtypes of IBD could contribute to a higher risk of IC, CD tended to play a stronger role in promoting the pathogenesis of IC. However, it is noteworthy that the risks of IC in CD and UC observed in observational studies were much higher than the risks observed in this MR study. One possible reason is that patients enrolled in observational studies often had one or more comorbidities rather than CD or UC alone, leading to an overestimation of IC risk. Currently, there are no large-sample cohort studies or RCT studies to investigate whether IC can promote the occurrence of CD and UC, which we hope can be explored in future studies.

As a common extraintestinal manifestation of IBD though, the pathogenesis of IC in IBD is still perplexing. However, an immune-mediated mechanism is postulated since both traditional glucocorticoids and newer biological therapy have been proven to treat IC, UC, and CD effectively [6,7,23,24,25]. Common antigens in the gut and the eye may also be responsible for the intestinal and ocular inflammation in IBD. For example, a shared and unique peptide, 7E12H12, has been found in both colon epithelial cells and non-pigmented ciliary epithelial cells [26]. Moreover, some cytokines, such as IL-6, IL-10, and IL-17, are proven to play a role in the pathogenesis of IC and two entities of IBD, suggesting that there may be a common immunopathogenesis [27,28]. Vitamin D deficiency may also play a common role in the development of IC, UC, and CD. Several studies have shown that vitamin D can suppress the increased activity of immune cells such as B and T lymphocytes by inhibiting their proliferation and differentiation, thus performing an anti-inflammatory action [29,30]. An inverse association between vitamin D levels and the development of IC, CD, and UC has also been demonstrated, indicating a protective role of higher vitamin D levels on IC, CD, and UC [29,30]. Most Vitamin D is synthesized in the skin after exposure to sunlight with only a small amount obtained from food intake, whereas insufficient sunlight exposure is a risk factor for all uveitis (including IC), UC, and CD [21,31,32]. The above phenomena suggest that there may be one or more immune-mediated mechanisms that provide a bridge between IBD and IC.

**Figure 5:**
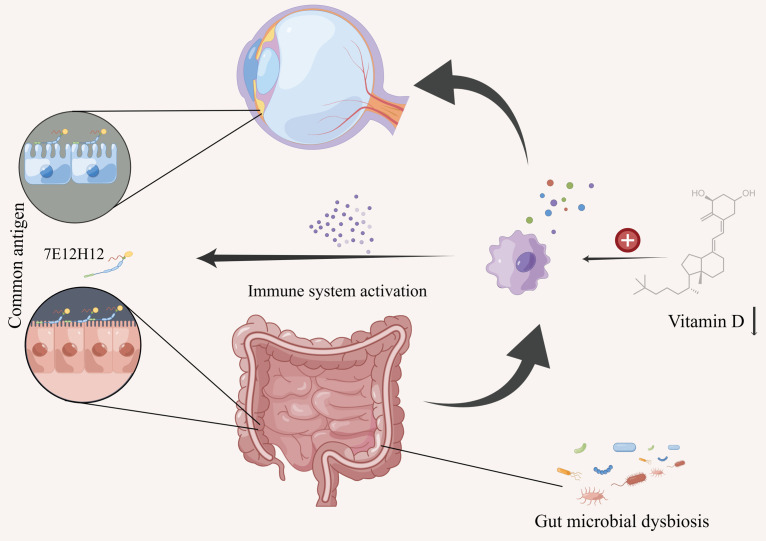
The possible mechanisms of iridocyclitis in inflammatory bowel disease. Different mechanisms, including shared peptide (common antigen), Vitamin D deficiency, and gut microbial dysbiosis could be involved. All of the mechanisms are immune-mediated, although other mechanisms may also exist.

Historically, UC and CD have always been studied together due to their similar features. However, it is now very clear that they represent two interconnected but different pathophysiological entities [28]. For example, CD may involve any part of the gastrointestinal tract, causing a wide variety of clinical presentations dictated by the location and severity of inflammation [33]. The inflammation of UC is confined to the colonic mucosa, delivering less heterogeneous presentations than CD [33]. In addition, 163 genetic loci have been recognized to implicate the development of IBD at present, among which 110 are associated with both forms of IBD, 30 are CD-specific, and 23 are UC-specific, so both differences and connections exist in the genetic susceptibility of CD and UC [34]. Moreover, it has been widely recognized that abnormal gut microbiota plays an important role in the development of IBD since many studies have observed significant differences in the gut microbiomes of IBD patients compared with healthy subjects [35,36]. However, a recent study indicates that the gut microbiome of UC patients is relatively closer to healthy subjects compared with CD, suggesting that gut microbial dysbiosis is disease-specific in the two entities of IBD [37]. Although subtle, all these distinctions may be the reason why CD has a stronger association with IC than UC. Still, it must be noted that all contributors to IBD, i.e., the immune system, genetic susceptibility, environment, and gut microbiota, should be taken into account when trying to understand the connection between IC and IBD [27,28,29,30,31,32,34,35,36,37,38,39,40]. Thus, other mechanisms are possible in place. The possible mechanisms of IC in IBD are summarized in Figure 5.

In the reverse direction of MR analysis, we found that patients with IC do not suffer a higher risk of UC or CD. Similar to other forms of uveitis, IC is often seen as a local manifestation of some systemic diseases [5]. However, whether IC can trigger systemic diseases such as IBD is unknown. In the present MR study, no association from IC to IBD was detected, but future real-world study is needed to strengthen or refute this finding.

There are three main strengths worth noting in the present study. First, the MR design enables this study to simulate an RCT study. RCTs are costly, time-consuming, and sometimes impractical to conduct. MR provides a solution to such shortcomings by investigating the causality in a low-cost and convenient way. Second, observational studies are inevitably affected by reverse causal effects, thus are unable to accurately infer the directionality of the causality. The bidirectional MR design in this study has solved this problem. Third, this is the first study that has performed an MR analysis to address the causality between IBD and IC. Considering that the incidence of IBD is soaring worldwide, revealing causality between IBD and IC will promote early diagnosis and intervention of high-risk populations.

This study has several limitations. First, we did not distinguish IBD and IC patients by gender, so the possible gender-specific effects on the association might be ignored. Moreover, we cannot completely rule out the possible effect of heterogeneity on the results although measures have been taken to identify and remove outlier SNPs. Additionally, our study has reported the causal relationship between IBD and IC, but the precise underlying mechanisms have not been well understood, so further research is warranted. Last, since the risk factors for IBD and IC include not only genetic factors but also other factors, such as environmental ones, confounding cannot be completely eliminated despite using an MR study design.

## 5. Conclusions

This is the first MR study to corroborate the associations between IBD and IC. We found that both CD and UC were associated with an increased risk of IC whereas CD tended to play a stronger role in promoting the pathogenesis of IC. In the reverse direction, IC seemed not to cause a higher risk of UC or CD. This paper emphasizes the importance of ophthalmic examinations for IBD patients, especially those with CD.

## Data Availability

The datasets for this study can be found in the GWAS database (https://gwas.mrcieu.ac.uk/) (accessed on 5 December 2022) and FinnGen database (http://www.finngen.fi) (accessed on 5 December 2022).

## Supplementary Materials

The following supporting information can be downloaded at: https://www.mdpi.com/article/10.3390/jcm12041282/s1, Table S1: SNPs for IBD; Table S2: SNPs for IC.
