# Supplementary material for: Association between Inflammatory Bowel Disease and Iridocyclitis: A Mendelian Randomization Study
Source: J Clin Med. 2023 Feb 6;12(4):1282. doi: 10.3390/jcm12041282 (PMC9960523; doi:10.3390/jcm12041282)
Supplement: Supplementary file 1 [file jcm-12-01282-s001.zip › jcm-2170209-supplementary/Supplementary files/Supplementary Material Table 1.docx]

Supplementary Material

Causal associations between inflammatory bowel disease and iridocyclitis: A Mendelian randomization study

## Supplementary Table 1

**SNPs related to IBD**

| **ID** | **SNP** | **Effect_allele** | **Other_allele** | **Se** | **Beta** | **Chr** | **P_val** | **F** |
| --- | --- | --- | --- | --- | --- | --- | --- | --- |
| ebi-a-GCST004131 | rs12136659 | C | T | 0.014 | 0.087 | 1 | 1.02E-09 | 37.537 |
| ebi-a-GCST004131 | rs2488398 | C | G | 0.015 | 0.099 | 1 | 3.63E-11 | 43.702 |
| ebi-a-GCST004131 | rs10746475 | A | T | 0.016 | 0.131 | 1 | 1.58E-15 | 63.610 |
| ebi-a-GCST004131 | rs4654925 | C | G | 0.012 | -0.117 | 1 | 4.80E-21 | 89.333 |
| ebi-a-GCST004131 | rs112936798 | C | A | 0.033 | -0.184 | 1 | 2.89E-08 | 30.849 |
| ebi-a-GCST004131 | rs35730213 | C | G | 0.014 | -0.135 | 1 | 7.50E-22 | 92.434 |
| ebi-a-GCST004131 | rs3024493 | A | C | 0.017 | 0.191 | 1 | 4.04E-31 | 134.139 |
| ebi-a-GCST004131 | rs11209013 | G | A | 0.012 | 0.077 | 1 | 4.46E-10 | 38.861 |
| ebi-a-GCST004131 | rs11581607 | A | G | 0.029 | -0.658 | 1 | 4.59E-111 | 500.603 |
| ebi-a-GCST004131 | rs1336900 | A | G | 0.013 | -0.085 | 1 | 2.98E-11 | 43.891 |
| ebi-a-GCST004131 | rs10800309 | G | A | 0.013 | -0.123 | 1 | 1.94E-20 | 85.528 |
| ebi-a-GCST004131 | rs1268339 | C | T | 0.016 | 0.091 | 1 | 2.75E-08 | 30.963 |
| ebi-a-GCST004131 | rs1317209 | A | G | 0.016 | 0.116 | 1 | 3.79E-13 | 52.926 |
| ebi-a-GCST004131 | rs3820330 | A | C | 0.014 | -0.089 | 1 | 1.72E-10 | 40.595 |
| ebi-a-GCST004131 | rs4276914 | A | G | 0.013 | 0.078 | 1 | 3.15E-10 | 39.238 |
| ebi-a-GCST004131 | rs7532133 | G | A | 0.013 | 0.079 | 1 | 3.83E-09 | 34.669 |
| ebi-a-GCST004131 | rs11677002 | C | T | 0.013 | -0.093 | 2 | 1.37E-13 | 54.596 |
| ebi-a-GCST004131 | rs55946629 | A | C | 0.018 | 0.130 | 2 | 5.45E-13 | 52.000 |
| ebi-a-GCST004131 | rs4676408 | A | G | 0.013 | 0.101 | 2 | 7.63E-15 | 60.481 |
| ebi-a-GCST004131 | rs7608697 | C | A | 0.013 | 0.140 | 2 | 1.67E-28 | 122.577 |
| ebi-a-GCST004131 | rs13422838 | C | T | 0.021 | -0.114 | 2 | 2.56E-08 | 31.087 |
| ebi-a-GCST004131 | rs62180107 | C | G | 0.013 | -0.080 | 2 | 1.55E-09 | 36.456 |
| ebi-a-GCST004131 | rs3792111 | T | C | 0.012 | 0.139 | 2 | 5.12E-29 | 125.838 |
| ebi-a-GCST004131 | rs1558619 | T | G | 0.012 | -0.084 | 2 | 8.90E-12 | 46.973 |
| ebi-a-GCST004131 | rs76286777 | C | T | 0.015 | 0.100 | 2 | 4.65E-11 | 43.508 |
| ebi-a-GCST004131 | rs72852162 | C | A | 0.020 | -0.113 | 2 | 2.30E-08 | 31.238 |
| ebi-a-GCST004131 | rs6740847 | G | A | 0.013 | -0.092 | 2 | 1.22E-13 | 54.642 |
| ebi-a-GCST004131 | rs62183956 | T | C | 0.013 | -0.078 | 2 | 4.49E-10 | 38.938 |
| ebi-a-GCST004131 | rs1131095 | C | T | 0.013 | 0.164 | 3 | 1.22E-35 | 155.773 |
| ebi-a-GCST004131 | rs56116661 | T | C | 0.016 | -0.100 | 3 | 9.27E-10 | 37.638 |
| ebi-a-GCST004131 | rs77272631 | C | G | 0.042 | 0.229 | 3 | 3.72E-08 | 30.237 |
| ebi-a-GCST004131 | rs503734 | G | A | 0.012 | -0.069 | 3 | 2.67E-08 | 31.144 |
| ebi-a-GCST004131 | rs2593855 | T | C | 0.014 | -0.083 | 3 | 2.54E-09 | 35.318 |
| ebi-a-GCST004131 | rs11734570 | A | G | 0.013 | 0.069 | 4 | 4.80E-08 | 29.861 |
| ebi-a-GCST004131 | rs62324212 | A | C | 0.013 | 0.089 | 4 | 2.67E-12 | 48.670 |
| ebi-a-GCST004131 | rs4957256 | T | C | 0.016 | -0.118 | 5 | 3.37E-14 | 57.858 |
| ebi-a-GCST004131 | rs17656349 | T | C | 0.013 | 0.073 | 5 | 5.17E-09 | 34.199 |
| ebi-a-GCST004131 | rs6579807 | T | C | 0.019 | 0.125 | 5 | 4.01E-11 | 43.742 |
| ebi-a-GCST004131 | rs1445004 | T | C | 0.013 | 0.169 | 5 | 3.48E-40 | 176.869 |
| ebi-a-GCST004131 | rs62378712 | C | T | 0.014 | -0.078 | 5 | 4.23E-08 | 29.864 |
| ebi-a-GCST004131 | rs6873866 | C | T | 0.013 | -0.092 | 5 | 6.15E-13 | 51.548 |
| ebi-a-GCST004131 | rs10041497 | C | T | 0.013 | 0.082 | 5 | 1.95E-10 | 40.308 |
| ebi-a-GCST004131 | rs755374 | T | C | 0.013 | 0.177 | 5 | 1.59E-39 | 173.886 |
| ebi-a-GCST004131 | rs56235845 | G | T | 0.014 | 0.088 | 5 | 1.77E-10 | 40.387 |
| ebi-a-GCST004131 | rs11739135 | C | G | 0.013 | 0.137 | 5 | 1.10E-27 | 119.421 |
| ebi-a-GCST004131 | rs341295 | T | C | 0.012 | 0.070 | 5 | 1.45E-08 | 32.050 |
| ebi-a-GCST004131 | rs11152949 | G | A | 0.013 | 0.102 | 6 | 1.56E-14 | 58.701 |
| ebi-a-GCST004131 | rs1267496 | C | G | 0.016 | 0.105 | 6 | 3.39E-11 | 43.859 |
| ebi-a-GCST004131 | rs145568234 | G | T | 0.048 | 0.860 | 6 | 4.73E-73 | 326.425 |
| ebi-a-GCST004131 | rs6457681 | T | G | 0.015 | -0.169 | 6 | 3.75E-28 | 121.576 |
| ebi-a-GCST004131 | rs4712528 | C | G | 0.015 | 0.104 | 6 | 7.14E-12 | 47.085 |
| ebi-a-GCST004131 | rs143210366 | G | T | 0.036 | 0.284 | 6 | 3.14E-15 | 62.059 |
| ebi-a-GCST004131 | rs62408218 | T | C | 0.013 | -0.082 | 6 | 2.40E-10 | 40.209 |
| ebi-a-GCST004131 | rs212402 | A | G | 0.013 | -0.074 | 6 | 1.06E-08 | 32.666 |
| ebi-a-GCST004131 | rs34140409 | T | C | 0.024 | -0.158 | 6 | 2.28E-11 | 44.613 |
| ebi-a-GCST004131 | rs6933404 | C | T | 0.015 | 0.086 | 6 | 6.64E-09 | 33.547 |
| ebi-a-GCST004131 | rs35171809 | G | A | 0.012 | 0.109 | 6 | 1.16E-18 | 78.243 |
| ebi-a-GCST004131 | rs10953551 | G | A | 0.013 | -0.103 | 7 | 4.94E-16 | 66.160 |
| ebi-a-GCST004131 | rs243505 | G | A | 0.013 | -0.081 | 7 | 3.04E-10 | 39.552 |
| ebi-a-GCST004131 | rs149169037 | A | G | 0.024 | -0.134 | 7 | 3.26E-08 | 30.569 |
| ebi-a-GCST004131 | rs1456896 | T | C | 0.013 | 0.088 | 7 | 4.50E-11 | 43.679 |
| ebi-a-GCST004131 | rs62482552 | A | G | 0.013 | -0.074 | 7 | 1.97E-08 | 31.651 |
| ebi-a-GCST004131 | rs11768365 | G | A | 0.015 | -0.084 | 7 | 3.88E-08 | 30.322 |
| ebi-a-GCST004131 | rs78771661 | T | C | 0.067 | -0.385 | 8 | 8.95E-09 | 33.084 |
| ebi-a-GCST004131 | rs4380956 | A | G | 0.013 | 0.091 | 8 | 1.12E-12 | 51.004 |
| ebi-a-GCST004131 | rs938650 | A | G | 0.019 | -0.107 | 8 | 1.41E-08 | 32.291 |
| ebi-a-GCST004131 | rs1887428 | C | G | 0.013 | -0.164 | 9 | 2.46E-36 | 157.301 |
| ebi-a-GCST004131 | rs10114470 | C | T | 0.014 | 0.148 | 9 | 4.10E-27 | 115.916 |
| ebi-a-GCST004131 | rs3829110 | G | A | 0.013 | 0.157 | 9 | 3.52E-36 | 158.558 |
| ebi-a-GCST004131 | rs1250573 | A | G | 0.014 | -0.098 | 10 | 1.11E-12 | 50.431 |
| ebi-a-GCST004131 | rs10826797 | T | G | 0.014 | -0.099 | 10 | 3.99E-13 | 52.990 |
| ebi-a-GCST004131 | rs6584282 | G | A | 0.012 | -0.152 | 10 | 1.19E-34 | 150.260 |
| ebi-a-GCST004131 | rs11195128 | T | C | 0.013 | 0.079 | 10 | 2.74E-09 | 35.461 |
| ebi-a-GCST004131 | rs2384352 | G | A | 0.013 | 0.095 | 10 | 3.12E-13 | 52.701 |
| ebi-a-GCST004131 | rs10761659 | G | A | 0.013 | 0.159 | 10 | 2.30E-36 | 158.240 |
| ebi-a-GCST004131 | rs7918084 | T | C | 0.013 | 0.071 | 10 | 1.38E-08 | 32.262 |
| ebi-a-GCST004131 | rs111456533 | A | G | 0.017 | -0.103 | 10 | 1.18E-09 | 36.781 |
| ebi-a-GCST004131 | rs11221335 | C | T | 0.015 | 0.083 | 11 | 2.44E-08 | 31.224 |
| ebi-a-GCST004131 | rs11236797 | A | C | 0.013 | 0.149 | 11 | 7.19E-33 | 141.705 |
| ebi-a-GCST004131 | rs11066188 | A | G | 0.013 | 0.087 | 12 | 1.76E-11 | 45.200 |
| ebi-a-GCST004131 | rs117981694 | A | G | 0.041 | 0.345 | 12 | 4.53E-17 | 70.544 |
| ebi-a-GCST004131 | rs12825700 | A | G | 0.013 | 0.132 | 12 | 1.27E-25 | 108.685 |
| ebi-a-GCST004131 | rs3897234 | C | T | 0.015 | 0.097 | 13 | 1.90E-11 | 44.844 |
| ebi-a-GCST004131 | rs140933577 | C | T | 0.031 | -0.186 | 13 | 1.13E-09 | 37.070 |
| ebi-a-GCST004131 | rs194746 | T | C | 0.012 | 0.083 | 14 | 1.84E-11 | 45.128 |
| ebi-a-GCST004131 | rs3850378 | C | T | 0.021 | 0.154 | 14 | 1.10E-13 | 55.061 |
| ebi-a-GCST004131 | rs1864239 | G | A | 0.178 | 1.337 | 15 | 6.27E-14 | 56.258 |
| ebi-a-GCST004131 | rs56062135 | T | C | 0.015 | 0.138 | 15 | 1.37E-21 | 90.841 |
| ebi-a-GCST004131 | rs7190426 | C | A | 0.016 | -0.087 | 16 | 2.06E-08 | 31.650 |
| ebi-a-GCST004131 | rs28374519 | A | G | 0.014 | -0.111 | 16 | 6.55E-16 | 65.055 |
| ebi-a-GCST004131 | rs9934775 | T | C | 0.017 | -0.112 | 16 | 8.77E-11 | 42.099 |
| ebi-a-GCST004131 | rs8056255 | A | T | 0.033 | 0.277 | 16 | 2.99E-17 | 71.498 |
| ebi-a-GCST004131 | rs11548656 | G | A | 0.036 | -0.237 | 16 | 5.18E-11 | 43.008 |
| ebi-a-GCST004131 | rs749910 | A | G | 0.014 | 0.196 | 16 | 7.83E-46 | 201.928 |
| ebi-a-GCST004131 | rs2301127 | A | G | 0.013 | 0.078 | 16 | 4.96E-10 | 38.617 |
| ebi-a-GCST004131 | rs16940202 | C | T | 0.017 | 0.113 | 16 | 2.50E-11 | 44.708 |
| ebi-a-GCST004131 | rs12936409 | T | C | 0.012 | 0.141 | 17 | 7.73E-30 | 128.566 |
| ebi-a-GCST004131 | rs744166 | G | A | 0.013 | -0.111 | 17 | 1.34E-18 | 77.468 |
| ebi-a-GCST004131 | rs714910 | C | A | 0.014 | -0.096 | 17 | 6.23E-12 | 47.600 |
| ebi-a-GCST004131 | rs113846785 | CG | C | 0.018 | -0.132 | 17 | 2.47E-13 | 53.696 |
| ebi-a-GCST004131 | rs1319951 | G | C | 0.015 | -0.085 | 18 | 7.50E-09 | 33.514 |
| ebi-a-GCST004131 | rs80262450 | A | G | 0.019 | 0.158 | 18 | 1.04E-16 | 69.240 |
| ebi-a-GCST004131 | rs4807569 | C | A | 0.015 | 0.128 | 19 | 4.24E-17 | 71.025 |
| ebi-a-GCST004131 | rs7256518 | A | G | 0.028 | -0.167 | 19 | 1.63E-09 | 36.392 |
| ebi-a-GCST004131 | rs62126610 | G | A | 0.017 | 0.141 | 19 | 2.60E-17 | 71.841 |
| ebi-a-GCST004131 | rs11669299 | T | C | 0.016 | -0.111 | 19 | 1.84E-12 | 49.716 |
| ebi-a-GCST004131 | rs6062496 | A | G | 0.013 | 0.137 | 20 | 2.83E-26 | 112.788 |
| ebi-a-GCST004131 | rs4256018 | G | T | 0.014 | 0.079 | 20 | 1.23E-08 | 32.440 |
| ebi-a-GCST004131 | rs6017342 | C | A | 0.014 | 0.116 | 20 | 1.07E-17 | 73.324 |
| ebi-a-GCST004131 | rs6063502 | G | A | 0.013 | -0.073 | 20 | 4.55E-08 | 30.004 |
| ebi-a-GCST004131 | rs154873 | A | G | 0.013 | -0.081 | 20 | 7.38E-10 | 37.934 |
| ebi-a-GCST004131 | rs1297264 | G | A | 0.013 | -0.146 | 21 | 3.98E-31 | 134.634 |
| ebi-a-GCST004131 | rs2836881 | T | G | 0.015 | -0.164 | 21 | 1.96E-29 | 126.640 |
| ebi-a-GCST004131 | rs2838517 | C | T | 0.013 | -0.128 | 21 | 1.83E-24 | 104.858 |
| ebi-a-GCST004131 | rs2413583 | T | C | 0.017 | -0.173 | 22 | 4.60E-24 | 102.590 |
| ebi-a-GCST004131 | rs5754100 | C | T | 0.016 | 0.129 | 22 | 7.14E-16 | 65.307 |
| ebi-a-GCST004131 | rs5763793 | T | G | 0.013 | 0.073 | 22 | 1.47E-08 | 31.879 |

**SNPs related to UC**

| **ID** | **SNP** | **Effect_allele** | **Other_allele** | **Se** | **Beta** | **Chr** | **P_val** | **F** |
| --- | --- | --- | --- | --- | --- | --- | --- | --- |
| ebi-a-GCST004133 | rs7544646 | G | C | 0.016 | -0.117 | 1 | 2.53E-13 | 53.290 |
| ebi-a-GCST004133 | rs3024493 | A | C | 0.021 | 0.210 | 1 | 7.46E-24 | 100.959 |
| ebi-a-GCST004133 | rs3820330 | A | C | 0.018 | -0.159 | 1 | 3.91E-19 | 79.490 |
| ebi-a-GCST004133 | rs11209026 | A | G | 0.036 | -0.483 | 1 | 1.99E-41 | 182.024 |
| ebi-a-GCST004133 | rs6658353 | C | G | 0.016 | -0.157 | 1 | 1.17E-22 | 96.163 |
| ebi-a-GCST004133 | rs7554511 | A | C | 0.018 | -0.145 | 1 | 4.27E-16 | 66.175 |
| ebi-a-GCST004133 | rs7523335 | A | G | 0.021 | -0.139 | 1 | 3.42E-11 | 43.749 |
| ebi-a-GCST004133 | rs2816954 | A | T | 0.023 | 0.138 | 1 | 1.80E-09 | 36.052 |
| ebi-a-GCST004133 | rs1317209 | A | G | 0.020 | 0.182 | 1 | 2.90E-19 | 80.204 |
| ebi-a-GCST004133 | rs79051659 | A | G | 0.026 | 0.161 | 1 | 1.30E-09 | 36.961 |
| ebi-a-GCST004133 | rs4654925 | C | G | 0.016 | -0.222 | 1 | 2.61E-44 | 194.418 |
| ebi-a-GCST004133 | rs7608697 | C | A | 0.016 | 0.160 | 2 | 3.03E-23 | 98.392 |
| ebi-a-GCST004133 | rs55905347 | A | G | 0.017 | 0.105 | 2 | 2.09E-10 | 40.315 |
| ebi-a-GCST004133 | rs62180181 | T | C | 0.017 | 0.123 | 2 | 8.08E-13 | 51.403 |
| ebi-a-GCST004133 | rs4676408 | A | G | 0.017 | 0.143 | 2 | 1.19E-17 | 73.631 |
| ebi-a-GCST004133 | rs1811711 | G | C | 0.022 | -0.130 | 2 | 6.09E-09 | 33.932 |
| ebi-a-GCST004133 | rs1131095 | C | T | 0.017 | 0.159 | 3 | 2.18E-21 | 89.911 |
| ebi-a-GCST004133 | rs755374 | T | C | 0.017 | 0.171 | 5 | 9.73E-24 | 100.468 |
| ebi-a-GCST004133 | rs72704802 | T | C | 0.021 | -0.122 | 5 | 2.89E-09 | 35.247 |
| ebi-a-GCST004133 | rs17715902 | A | G | 0.017 | 0.097 | 5 | 4.62E-09 | 34.427 |
| ebi-a-GCST004133 | rs6889364 | A | G | 0.023 | 0.132 | 5 | 7.87E-09 | 33.417 |
| ebi-a-GCST004133 | rs17656349 | T | C | 0.016 | 0.090 | 5 | 1.54E-08 | 32.040 |
| ebi-a-GCST004133 | rs67111717 | G | A | 0.017 | 0.094 | 5 | 3.27E-08 | 30.476 |
| ebi-a-GCST004133 | rs9260809 | G | A | 0.018 | -0.110 | 6 | 1.96E-09 | 35.870 |
| ebi-a-GCST004133 | rs9267798 | C | G | 0.028 | 0.249 | 6 | 6.54E-19 | 78.829 |
| ebi-a-GCST004133 | rs9271176 | G | A | 0.017 | -0.350 | 6 | 4.20E-91 | 408.133 |
| ebi-a-GCST004133 | rs28383224 | G | A | 0.017 | -0.147 | 6 | 4.65E-19 | 79.156 |
| ebi-a-GCST004133 | rs3734851 | A | G | 0.058 | 0.503 | 6 | 6.58E-18 | 74.273 |
| ebi-a-GCST004133 | rs13200059 | A | G | 0.044 | 0.294 | 6 | 1.48E-11 | 45.593 |
| ebi-a-GCST004133 | rs6933404 | C | T | 0.019 | 0.149 | 6 | 2.69E-15 | 62.477 |
| ebi-a-GCST004133 | rs113986290 | T | C | 0.053 | -0.307 | 6 | 7.59E-09 | 33.339 |
| ebi-a-GCST004133 | rs798506 | C | T | 0.018 | -0.121 | 7 | 1.47E-11 | 45.393 |
| ebi-a-GCST004133 | rs4728142 | A | G | 0.016 | 0.100 | 7 | 3.23E-10 | 39.658 |
| ebi-a-GCST004133 | rs989960 | T | C | 0.016 | -0.121 | 7 | 3.28E-14 | 57.570 |
| ebi-a-GCST004133 | rs10272963 | T | C | 0.016 | -0.151 | 7 | 4.11E-21 | 89.303 |
| ebi-a-GCST004133 | rs1887428 | C | G | 0.017 | -0.167 | 9 | 9.65E-24 | 101.208 |
| ebi-a-GCST004133 | rs10817678 | A | G | 0.017 | 0.133 | 9 | 4.42E-15 | 61.392 |
| ebi-a-GCST004133 | rs3812565 | C | T | 0.016 | 0.134 | 9 | 6.50E-17 | 69.618 |
| ebi-a-GCST004133 | rs10761659 | G | A | 0.016 | 0.128 | 10 | 1.33E-15 | 63.601 |
| ebi-a-GCST004133 | rs7911117 | G | T | 0.024 | -0.134 | 10 | 1.84E-08 | 31.529 |
| ebi-a-GCST004133 | rs7911680 | C | A | 0.016 | -0.153 | 10 | 6.71E-22 | 91.991 |
| ebi-a-GCST004133 | rs2212434 | T | C | 0.016 | 0.125 | 11 | 2.80E-15 | 62.003 |
| ebi-a-GCST004133 | rs2045241 | A | G | 0.017 | -0.106 | 11 | 2.83E-10 | 39.563 |
| ebi-a-GCST004133 | rs12825700 | A | G | 0.016 | 0.189 | 12 | 7.33E-32 | 137.661 |
| ebi-a-GCST004133 | rs1359946 | A | G | 0.020 | 0.157 | 13 | 6.58E-15 | 60.485 |
| ebi-a-GCST004133 | rs56062135 | T | C | 0.018 | 0.108 | 15 | 4.66E-09 | 34.324 |
| ebi-a-GCST004133 | rs11645239 | G | C | 0.020 | -0.117 | 16 | 4.14E-09 | 34.457 |
| ebi-a-GCST004133 | rs7203363 | A | T | 0.019 | 0.107 | 16 | 1.41E-08 | 32.111 |
| ebi-a-GCST004133 | rs16940186 | C | T | 0.021 | 0.136 | 16 | 2.18E-10 | 40.210 |
| ebi-a-GCST004133 | rs113846785 | CG | C | 0.023 | -0.163 | 17 | 1.15E-12 | 50.478 |
| ebi-a-GCST004133 | rs12936409 | T | C | 0.016 | 0.137 | 17 | 5.62E-18 | 74.636 |
| ebi-a-GCST004133 | rs11651246 | G | T | 0.022 | 0.147 | 17 | 2.01E-11 | 45.055 |
| ebi-a-GCST004133 | rs10408351 | A | G | 0.020 | 0.155 | 19 | 2.92E-14 | 57.581 |
| ebi-a-GCST004133 | rs78064630 | A | G | 0.031 | 0.176 | 19 | 1.08E-08 | 32.616 |
| ebi-a-GCST004133 | rs6062496 | A | G | 0.016 | 0.136 | 20 | 8.97E-17 | 69.513 |
| ebi-a-GCST004133 | rs6017342 | C | A | 0.017 | 0.194 | 20 | 3.95E-30 | 130.766 |
| ebi-a-GCST004133 | rs2836881 | T | G | 0.019 | -0.222 | 21 | 1.11E-32 | 142.071 |
| ebi-a-GCST004133 | rs2838517 | C | T | 0.016 | -0.118 | 21 | 1.78E-13 | 54.114 |
| ebi-a-GCST004133 | rs1736161 | A | G | 0.016 | -0.123 | 21 | 2.22E-14 | 58.081 |
| ebi-a-GCST004133 | rs9611131 | C | T | 0.023 | -0.149 | 22 | 5.11E-11 | 43.316 |
| ebi-a-GCST004133 | rs4993442 | T | G | 0.018 | -0.099 | 22 | 3.54E-08 | 30.465 |
| ebi-a-GCST004133 | rs137845 | G | A | 0.016 | 0.101 | 22 | 1.50E-10 | 40.944 |

**SNPs related to CD**

| **ID** | **SNP** | **Effect_allele** | **Other_allele** | **Se** | **Beta** | **Chr** | **P_val** | **F** |
| --- | --- | --- | --- | --- | --- | --- | --- | --- |
| ebi-a-GCST004132 | rs12131079 | T | C | 0.017 | -0.109 | 1 | 3.99E-10 | 39.098 |
| ebi-a-GCST004132 | rs35730213 | C | G | 0.018 | -0.117 | 1 | 1.17E-10 | 41.499 |
| ebi-a-GCST004132 | rs3122605 | A | G | 0.023 | -0.175 | 1 | 1.24E-14 | 59.297 |
| ebi-a-GCST004132 | rs114802258 | T | C | 0.038 | -0.225 | 1 | 5.11E-09 | 34.180 |
| ebi-a-GCST004132 | rs4316387 | C | T | 0.019 | -0.129 | 1 | 7.74E-12 | 46.731 |
| ebi-a-GCST004132 | rs6679677 | A | C | 0.029 | -0.228 | 1 | 1.77E-15 | 63.275 |
| ebi-a-GCST004132 | rs6704109 | T | C | 0.018 | 0.175 | 1 | 5.10E-22 | 93.267 |
| ebi-a-GCST004132 | rs7517847 | G | T | 0.017 | -0.345 | 1 | 5.84E-97 | 436.430 |
| ebi-a-GCST004132 | rs11378157 | AG | A | 0.019 | -0.138 | 2 | 9.29E-14 | 55.321 |
| ebi-a-GCST004132 | rs11683692 | C | T | 0.038 | -0.214 | 2 | 1.75E-08 | 31.833 |
| ebi-a-GCST004132 | rs4343432 | G | A | 0.016 | 0.112 | 2 | 3.50E-12 | 48.054 |
| ebi-a-GCST004132 | rs11677002 | C | T | 0.016 | -0.112 | 2 | 4.57E-12 | 47.551 |
| ebi-a-GCST004132 | rs34004493 | G | A | 0.018 | 0.126 | 2 | 2.00E-12 | 49.392 |
| ebi-a-GCST004132 | rs3816234 | A | G | 0.016 | 0.270 | 2 | 1.51E-62 | 278.601 |
| ebi-a-GCST004132 | rs55946629 | A | C | 0.023 | 0.176 | 2 | 2.85E-14 | 57.721 |
| ebi-a-GCST004132 | rs7608697 | C | A | 0.016 | 0.123 | 2 | 4.03E-14 | 56.850 |
| ebi-a-GCST004132 | rs6740847 | G | A | 0.016 | -0.104 | 2 | 9.72E-11 | 41.727 |
| ebi-a-GCST004132 | rs1583792 | T | C | 0.016 | -0.088 | 2 | 3.26E-08 | 30.388 |
| ebi-a-GCST004132 | rs56116661 | T | C | 0.021 | -0.131 | 3 | 5.67E-10 | 38.300 |
| ebi-a-GCST004132 | rs6808936 | G | A | 0.016 | 0.090 | 3 | 1.93E-08 | 31.527 |
| ebi-a-GCST004132 | rs9836291 | A | G | 0.017 | 0.172 | 3 | 3.77E-24 | 102.605 |
| ebi-a-GCST004132 | rs2581828 | G | C | 0.016 | -0.094 | 3 | 6.46E-09 | 33.740 |
| ebi-a-GCST004132 | rs73243877 | G | A | 0.021 | 0.116 | 4 | 4.12E-08 | 30.146 |
| ebi-a-GCST004132 | rs13107325 | T | C | 0.028 | 0.201 | 4 | 1.66E-12 | 49.891 |
| ebi-a-GCST004132 | rs62324212 | A | C | 0.016 | 0.106 | 4 | 8.02E-11 | 42.290 |
| ebi-a-GCST004132 | rs6579807 | T | C | 0.024 | 0.199 | 5 | 3.44E-16 | 66.717 |
| ebi-a-GCST004132 | rs755374 | T | C | 0.017 | 0.197 | 5 | 1.38E-29 | 128.054 |
| ebi-a-GCST004132 | rs6451494 | C | T | 0.017 | 0.261 | 5 | 8.26E-56 | 246.263 |
| ebi-a-GCST004132 | rs112856973 | C | T | 0.024 | -0.161 | 5 | 3.61E-11 | 44.007 |
| ebi-a-GCST004132 | rs6873866 | C | T | 0.016 | -0.131 | 5 | 1.35E-15 | 64.195 |
| ebi-a-GCST004132 | rs2188962 | T | C | 0.016 | 0.200 | 5 | 5.59E-36 | 156.876 |
| ebi-a-GCST004132 | rs181826 | A | C | 0.017 | 0.116 | 5 | 3.24E-12 | 48.415 |
| ebi-a-GCST004132 | rs1012636 | T | G | 0.020 | 0.129 | 6 | 7.01E-11 | 42.513 |
| ebi-a-GCST004132 | rs1321859 | T | C | 0.017 | -0.105 | 6 | 1.18E-09 | 37.196 |
| ebi-a-GCST004132 | rs73516754 | C | A | 0.017 | 0.142 | 6 | 4.04E-17 | 70.898 |
| ebi-a-GCST004132 | rs35171809 | G | A | 0.016 | 0.157 | 6 | 9.07E-23 | 97.004 |
| ebi-a-GCST004132 | rs111281598 | C | T | 0.032 | 0.275 | 6 | 4.17E-18 | 75.459 |
| ebi-a-GCST004132 | rs6941902 | C | T | 0.027 | 0.163 | 6 | 2.39E-09 | 35.649 |
| ebi-a-GCST004132 | rs7753014 | G | C | 0.016 | -0.099 | 6 | 1.39E-09 | 36.814 |
| ebi-a-GCST004132 | rs145568234 | G | T | 0.063 | 0.860 | 6 | 4.31E-42 | 184.668 |
| ebi-a-GCST004132 | rs9482770 | C | T | 0.016 | 0.099 | 6 | 1.01E-09 | 37.120 |
| ebi-a-GCST004132 | rs9501641 | T | C | 0.043 | 0.303 | 6 | 2.57E-12 | 49.097 |
| ebi-a-GCST004132 | rs9258357 | C | T | 0.022 | -0.118 | 6 | 5.00E-08 | 29.793 |
| ebi-a-GCST004132 | rs212409 | A | G | 0.016 | -0.110 | 6 | 1.49E-11 | 45.771 |
| ebi-a-GCST004132 | rs9656588 | C | T | 0.017 | 0.118 | 7 | 8.73E-12 | 46.760 |
| ebi-a-GCST004132 | rs938650 | A | G | 0.025 | -0.175 | 8 | 1.65E-12 | 50.026 |
| ebi-a-GCST004132 | rs4380956 | A | G | 0.017 | 0.132 | 8 | 1.15E-15 | 64.000 |
| ebi-a-GCST004132 | rs79832570 | C | T | 0.034 | 0.223 | 8 | 8.90E-11 | 42.174 |
| ebi-a-GCST004132 | rs10114470 | C | T | 0.018 | 0.169 | 9 | 1.76E-21 | 90.841 |
| ebi-a-GCST004132 | rs1887428 | C | G | 0.017 | -0.166 | 9 | 8.54E-23 | 96.481 |
| ebi-a-GCST004132 | rs4077515 | T | C | 0.016 | 0.185 | 9 | 3.14E-30 | 130.129 |
| ebi-a-GCST004132 | rs10884966 | A | G | 0.017 | 0.113 | 10 | 4.13E-11 | 43.745 |
| ebi-a-GCST004132 | rs61839660 | T | C | 0.026 | 0.147 | 10 | 1.98E-08 | 31.635 |
| ebi-a-GCST004132 | rs2002695 | G | A | 0.019 | -0.129 | 10 | 8.31E-12 | 46.803 |
| ebi-a-GCST004132 | rs10822050 | C | T | 0.016 | 0.183 | 10 | 2.35E-29 | 127.188 |
| ebi-a-GCST004132 | rs2675670 | C | G | 0.016 | 0.107 | 10 | 2.89E-11 | 44.500 |
| ebi-a-GCST004132 | rs1148246 | T | C | 0.017 | -0.132 | 10 | 2.09E-15 | 62.761 |
| ebi-a-GCST004132 | rs1250573 | A | G | 0.018 | -0.152 | 10 | 1.92E-17 | 72.297 |
| ebi-a-GCST004132 | rs6584282 | G | A | 0.016 | -0.166 | 10 | 3.44E-25 | 107.381 |
| ebi-a-GCST004132 | rs11236797 | A | C | 0.016 | 0.176 | 11 | 8.51E-28 | 119.502 |
| ebi-a-GCST004132 | rs28999107 | T | G | 0.018 | 0.108 | 12 | 1.06E-09 | 37.018 |
| ebi-a-GCST004132 | rs77566919 | A | G | 0.019 | -0.109 | 12 | 4.13E-09 | 34.651 |
| ebi-a-GCST004132 | rs34635748 | T | C | 0.050 | 0.479 | 12 | 1.95E-21 | 90.476 |
| ebi-a-GCST004132 | rs1373904 | G | A | 0.019 | 0.141 | 13 | 9.11E-14 | 55.656 |
| ebi-a-GCST004132 | rs194746 | T | C | 0.016 | 0.098 | 14 | 1.24E-09 | 36.674 |
| ebi-a-GCST004132 | rs3850378 | C | T | 0.027 | 0.199 | 14 | 8.31E-14 | 55.550 |
| ebi-a-GCST004132 | rs72743461 | A | C | 0.019 | 0.168 | 15 | 2.26E-19 | 81.096 |
| ebi-a-GCST004132 | rs2021511 | T | C | 0.018 | -0.108 | 16 | 2.63E-09 | 35.344 |
| ebi-a-GCST004132 | rs42861 | G | A | 0.017 | 0.124 | 16 | 8.87E-14 | 55.400 |
| ebi-a-GCST004132 | rs2076756 | G | A | 0.017 | 0.385 | 16 | 1.80E-108 | 489.579 |
| ebi-a-GCST004132 | rs7195228 | G | C | 0.021 | -0.133 | 16 | 2.09E-10 | 40.313 |
| ebi-a-GCST004132 | rs72798422 | C | T | 0.038 | 0.550 | 16 | 6.05E-47 | 206.923 |
| ebi-a-GCST004132 | rs10492862 | A | C | 0.018 | 0.107 | 16 | 1.26E-09 | 36.754 |
| ebi-a-GCST004132 | rs2948542 | G | A | 0.016 | 0.102 | 17 | 5.15E-10 | 38.852 |
| ebi-a-GCST004132 | rs714910 | C | A | 0.018 | -0.153 | 17 | 2.49E-17 | 71.547 |
| ebi-a-GCST004132 | rs12936409 | T | C | 0.016 | 0.143 | 17 | 4.31E-19 | 79.433 |
| ebi-a-GCST004132 | rs744166 | G | A | 0.016 | -0.114 | 17 | 1.80E-12 | 49.694 |
| ebi-a-GCST004132 | rs80262450 | A | G | 0.024 | 0.227 | 18 | 1.34E-20 | 86.399 |
| ebi-a-GCST004132 | rs144309607 | T | C | 0.047 | -0.371 | 19 | 2.69E-15 | 62.376 |
| ebi-a-GCST004132 | rs62126620 | A | G | 0.020 | 0.144 | 19 | 8.61E-13 | 51.325 |
| ebi-a-GCST004132 | rs4807570 | A | G | 0.019 | 0.181 | 19 | 6.03E-21 | 88.049 |
| ebi-a-GCST004132 | rs492602 | G | A | 0.016 | 0.108 | 19 | 2.33E-11 | 44.774 |
| ebi-a-GCST004132 | rs6062496 | A | G | 0.017 | 0.122 | 20 | 2.62E-13 | 53.632 |
| ebi-a-GCST004132 | rs3761158 | A | G | 0.017 | -0.110 | 20 | 2.65E-11 | 44.283 |
| ebi-a-GCST004132 | rs1297264 | G | A | 0.016 | -0.177 | 21 | 1.59E-27 | 117.782 |
| ebi-a-GCST004132 | rs2284553 | G | A | 0.017 | 0.128 | 21 | 1.14E-14 | 59.898 |
| ebi-a-GCST004132 | rs2838517 | C | T | 0.016 | -0.146 | 21 | 2.03E-19 | 80.778 |
| ebi-a-GCST004132 | rs2143178 | C | T | 0.022 | -0.209 | 22 | 6.84E-21 | 87.586 |
| ebi-a-GCST004132 | rs5754100 | C | T | 0.021 | 0.169 | 22 | 3.02E-16 | 67.065 |
