# Supplementary material for: Association between Inflammatory Bowel Disease and Iridocyclitis: A Mendelian Randomization Study
Source: J Clin Med. 2023 Feb 6;12(4):1282. doi: 10.3390/jcm12041282 (PMC9960523; doi:10.3390/jcm12041282)
Supplement: Supplementary file 1 [file jcm-12-01282-s001.zip › jcm-2170209-supplementary/Supplementary files/Supplementary Material Table 2.docx]

Supplementary Material

Causal associations between inflammatory bowel disease and iridocyclitis: A Mendelian randomization study

## Supplementary Table 2

**SNPs related to IRIDOCYCLITIS**

| **ID** | **SNP** | **Effect_allele** | **Other_allele** | **Se** | **Beta** | **P_val** | **F** |
| --- | --- | --- | --- | --- | --- | --- | --- |
| finngen_R7_H7_IRIDOCYCLITIS | rs7063 | T | A | 0.022 | -0.189 | 1.70E-17 | 72.468 |
| finngen_R7_H7_IRIDOCYCLITIS | rs34951809 | T | A | 0.039 | 0.324 | 1.65E-16 | 67.988 |
| finngen_R7_H7_IRIDOCYCLITIS | rs72833065 | C | T | 0.050 | 0.359 | 7.42E-13 | 51.430 |
| finngen_R7_H7_IRIDOCYCLITIS | rs2473167 | G | A | 0.038 | -0.456 | 2.99E-33 | 144.339 |
| finngen_R7_H7_IRIDOCYCLITIS | rs9393649 | T | C | 0.024 | 0.277 | 1.09E-29 | 128.056 |
| finngen_R7_H7_IRIDOCYCLITIS | rs17540043 | G | A | 0.044 | 0.449 | 1.95E-24 | 104.073 |
| finngen_R7_H7_IRIDOCYCLITIS | rs139591095 | C | T | 0.039 | 0.696 | 1.07E-70 | 316.006 |
| finngen_R7_H7_IRIDOCYCLITIS | rs147409637 | T | C | 0.041 | 0.807 | 5.10E-88 | 395.558 |
| finngen_R7_H7_IRIDOCYCLITIS | rs115537782 | A | G | 0.047 | 0.597 | 1.09E-36 | 160.072 |
| finngen_R7_H7_IRIDOCYCLITIS | rs184951744 | C | T | 0.033 | 0.923 | 1.99E-176 | 801.997 |
| finngen_R7_H7_IRIDOCYCLITIS | rs2524035 | G | A | 0.022 | 0.150 | 3.26E-12 | 48.526 |
| finngen_R7_H7_IRIDOCYCLITIS | rs2072109 | G | C | 0.032 | 0.557 | 4.84E-66 | 294.645 |
| finngen_R7_H7_IRIDOCYCLITIS | rs144835716 | T | A | 0.037 | 1.014 | 3.34E-164 | 745.772 |
| finngen_R7_H7_IRIDOCYCLITIS | rs2233941 | C | G | 0.031 | 0.943 | 1.00E-200 | 933.050 |
| finngen_R7_H7_IRIDOCYCLITIS | rs75456009 | G | A | 0.025 | -0.289 | 5.74E-32 | 138.474 |
| finngen_R7_H7_IRIDOCYCLITIS | rs2844557 | G | C | 0.019 | -0.336 | 4.72E-70 | 313.057 |
| finngen_R7_H7_IRIDOCYCLITIS | rs139776498 | T | C | 0.039 | 1.033 | 5.02E-151 | 685.164 |
| finngen_R7_H7_IRIDOCYCLITIS | rs9273539 | T | G | 0.029 | -0.234 | 1.11E-15 | 64.218 |
| finngen_R7_H7_IRIDOCYCLITIS | rs144112342 | C | T | 0.056 | 0.782 | 1.76E-44 | 195.763 |
| finngen_R7_H7_IRIDOCYCLITIS | rs9277559 | C | T | 0.023 | 0.227 | 5.96E-23 | 97.300 |
| finngen_R7_H7_IRIDOCYCLITIS | rs2281916 | C | T | 0.020 | 0.229 | 6.80E-31 | 133.565 |
| finngen_R7_H7_IRIDOCYCLITIS | rs116997897 | G | C | 0.049 | 0.947 | 1.11E-81 | 366.442 |
| finngen_R7_H7_IRIDOCYCLITIS | rs6902545 | A | G | 0.020 | -0.114 | 2.48E-08 | 31.076 |
| finngen_R7_H7_IRIDOCYCLITIS | rs74853132 | T | C | 0.048 | 0.428 | 3.49E-19 | 80.138 |
| finngen_R7_H7_IRIDOCYCLITIS | rs16884650 | G | A | 0.035 | 0.263 | 4.93E-14 | 56.757 |
| finngen_R7_H7_IRIDOCYCLITIS | rs181316459 | C | G | 0.042 | 0.375 | 4.46E-19 | 79.655 |

**SNPs related to IRIDOACUTE**

| **ID** | **SNP** | **Effect_allele** | **Other_allele** | **Se** | **Beta** | **P_val** | **F** |
| --- | --- | --- | --- | --- | --- | --- | --- |
| finngen_R7_H7_IRIDOACUTE | rs7535723 | T | C | 0.024 | -0.137 | 8.83E-09 | 33.082 |
| finngen_R7_H7_IRIDOACUTE | rs7063 | T | A | 0.024 | -0.188 | 3.57E-15 | 61.923 |
| finngen_R7_H7_IRIDOACUTE | rs34951809 | T | A | 0.041 | 0.390 | 5.53E-21 | 88.332 |
| finngen_R7_H7_IRIDOACUTE | rs72833065 | C | T | 0.053 | 0.411 | 9.61E-15 | 59.975 |
| finngen_R7_H7_IRIDOACUTE | rs2473167 | G | A | 0.040 | -0.497 | 5.98E-35 | 152.116 |
| finngen_R7_H7_IRIDOACUTE | rs9393649 | T | C | 0.026 | 0.319 | 2.26E-34 | 149.477 |
| finngen_R7_H7_IRIDOACUTE | rs9358888 | C | T | 0.021 | -0.118 | 3.06E-08 | 30.671 |
| finngen_R7_H7_IRIDOACUTE | rs17540043 | G | A | 0.047 | 0.475 | 4.16E-24 | 102.570 |
| finngen_R7_H7_IRIDOACUTE | rs139591095 | C | T | 0.041 | 0.760 | 3.52E-76 | 341.189 |
| finngen_R7_H7_IRIDOACUTE | rs145168562 | C | G | 0.043 | 0.871 | 3.33E-93 | 419.380 |
| finngen_R7_H7_IRIDOACUTE | rs115537782 | A | G | 0.050 | 0.644 | 7.14E-38 | 165.492 |
| finngen_R7_H7_IRIDOACUTE | rs184951744 | C | T | 0.034 | 0.990 | 4.11E-184 | 837.339 |
| finngen_R7_H7_IRIDOACUTE | rs2524035 | G | A | 0.023 | 0.164 | 1.71E-12 | 49.786 |
| finngen_R7_H7_IRIDOACUTE | rs2072109 | G | C | 0.035 | 0.600 | 1.09E-67 | 302.206 |
| finngen_R7_H7_IRIDOACUTE | rs144835716 | T | A | 0.039 | 1.082 | 2.76E-169 | 769.141 |
| finngen_R7_H7_IRIDOACUTE | rs2233941 | C | G | 0.032 | 1.007 | 1.00E-200 | 962.789 |
| finngen_R7_H7_IRIDOACUTE | rs75456009 | G | A | 0.027 | -0.332 | 1.19E-35 | 155.315 |
| finngen_R7_H7_IRIDOACUTE | rs2844560 | G | A | 0.020 | -0.370 | 2.42E-73 | 328.162 |
| finngen_R7_H7_IRIDOACUTE | rs554680493 | T | C | 0.030 | 0.525 | 1.46E-67 | 301.621 |
| finngen_R7_H7_IRIDOACUTE | rs6457642 | T | C | 0.026 | -0.253 | 1.08E-21 | 91.563 |
| finngen_R7_H7_IRIDOACUTE | rs151102334 | T | C | 0.040 | 0.262 | 5.97E-11 | 42.830 |
| finngen_R7_H7_IRIDOACUTE | rs147725790 | C | T | 0.036 | 0.949 | 1.72E-151 | 687.312 |
| finngen_R7_H7_IRIDOACUTE | rs7764080 | T | C | 0.029 | -0.205 | 2.84E-12 | 48.795 |
| finngen_R7_H7_IRIDOACUTE | rs140602536 | T | C | 0.039 | 0.456 | 2.76E-31 | 135.353 |
| finngen_R7_H7_IRIDOACUTE | rs74853132 | T | C | 0.051 | 0.470 | 1.98E-20 | 85.809 |
| finngen_R7_H7_IRIDOACUTE | rs75477235 | A | G | 0.068 | 0.373 | 4.24E-08 | 30.035 |
| finngen_R7_H7_IRIDOACUTE | rs114787942 | C | G | 0.040 | 0.313 | 4.97E-15 | 61.273 |
| finngen_R7_H7_IRIDOACUTE | rs181316459 | C | G | 0.044 | 0.446 | 7.66E-24 | 101.362 |

**SNPs related to IRIDOCHRONIC**

| **ID** | **SNP** | **Effect_allele** | **Other_allele** | **Se** | **Beta** | **P_val** | **F** |
| --- | --- | --- | --- | --- | --- | --- | --- |
| finn-b-H7_IRIDOCHRONIC | rs7762959 | C | T | 0.064 | 0.393 | 1.07E-09 | 37.186 |
| finn-b-H7_IRIDOCHRONIC | rs139591095 | T | C | 0.087 | 0.757 | 3.52E-18 | 75.576 |
| finn-b-H7_IRIDOCHRONIC | rs147409637 | C | T | 0.091 | 0.870 | 7.95E-22 | 92.172 |
| finn-b-H7_IRIDOCHRONIC | rs192247183 | G | C | 0.074 | 0.948 | 6.17E-38 | 165.784 |
| finn-b-H7_IRIDOCHRONIC | rs13195617 | A | T | 0.046 | 0.336 | 3.99E-13 | 52.646 |
| finn-b-H7_IRIDOCHRONIC | rs6913382 | T | C | 0.077 | 0.811 | 1.22E-25 | 109.561 |
| finn-b-H7_IRIDOCHRONIC | rs1264420 | A | G | 0.044 | 0.268 | 1.52E-09 | 36.506 |
| finn-b-H7_IRIDOCHRONIC | rs2517554 | C | T | 0.043 | -0.238 | 3.48E-08 | 30.419 |
| finn-b-H7_IRIDOCHRONIC | rs28752898 | A | G | 0.044 | 0.321 | 5.04E-13 | 52.190 |
| finn-b-H7_IRIDOCHRONIC | rs189600525 | C | T | 0.056 | 1.105 | 8.37E-86 | 385.386 |
| finn-b-H7_IRIDOCHRONIC | rs652888 | A | G | 0.048 | 0.402 | 7.81E-17 | 69.458 |
| finn-b-H7_IRIDOCHRONIC | rs148838821 | G | T | 0.083 | 0.970 | 1.82E-31 | 136.187 |
| finn-b-H7_IRIDOCHRONIC | rs9275500 | C | T | 0.044 | 0.282 | 1.43E-10 | 41.116 |
| finn-b-H7_IRIDOCHRONIC | rs2296329 | A | G | 0.047 | 0.292 | 5.60E-10 | 38.455 |
| finn-b-H7_IRIDOCHRONIC | rs116997897 | C | G | 0.115 | 0.911 | 2.20E-15 | 62.881 |

**SNPs related to IRIDONAS**

| **ID** | **SNP** | **Effect_allele** | **Other_allele** | **Se** | **Beta** | **P_val** | **F** |
| --- | --- | --- | --- | --- | --- | --- | --- |
| finn-b-H7_IRIDONAS | rs35482035 | A | G | 0.126 | 0.740 | 4.37E-09 | 34.453 |
| finn-b-H7_IRIDONAS | rs6910227 | A | G | 0.067 | 0.451 | 1.83E-11 | 45.140 |
| finn-b-H7_IRIDONAS | rs183044230 | G | C | 0.090 | 0.846 | 3.83E-21 | 89.059 |
| finn-b-H7_IRIDONAS | rs4713276 | G | C | 0.052 | 0.320 | 5.53E-10 | 38.481 |
| finn-b-H7_IRIDONAS | rs9391805 | T | C | 0.097 | 0.644 | 3.27E-11 | 44.011 |
| finn-b-H7_IRIDONAS | rs116488202 | T | C | 0.070 | 1.004 | 3.07E-46 | 203.817 |
| finn-b-H7_IRIDONAS | rs9391731 | A | C | 0.113 | 0.628 | 3.14E-08 | 30.616 |
| finn-b-H7_IRIDONAS | rs554680493 | T | C | 0.077 | 0.460 | 2.96E-09 | 35.212 |
| finn-b-H7_IRIDONAS | rs147725790 | C | T | 0.095 | 0.821 | 5.28E-18 | 74.773 |
